# Validation of the FEEL-KJ: An Instrument to Measure Emotion Regulation Strategies in Children and Adolescents

**DOI:** 10.1371/journal.pone.0137080

**Published:** 2015-09-02

**Authors:** Emiel Cracco, Kim Van Durme, Caroline Braet

**Affiliations:** 1 Department of Experimental Psychology, Ghent University, Ghent, Belgium; 2 Department of Developmental, Personality and Social Psychology, Ghent University, Ghent, Belgium; Tilburg University, NETHERLANDS

## Abstract

Although the field of emotion regulation in children and adolescents is growing, there is need for age-adjusted measures that assess a large variety of strategies. An interesting instrument in this respect is the FEEL-KJ because it measures 7 adaptive and 5 maladaptive emotion regulation strategies in response to three different emotions. However, the FEEL-KJ has not yet been validated extensively. Therefore, the current study aims to test the internal structure and validity of the FEEL-KJ in a large sample of Dutch-speaking Belgian children and adolescents (N = 1102, 8–18 years old). The investigation of the internal structure confirms earlier reports of a two-factor structure with Adaptive and Maladaptive Emotion Regulation as overarching categories. However, it also suggests that the two-factor model is more complex than what was previously assumed. The evaluation of the FEEL-KJ validity furthermore provides evidence for its construct and external validity. In sum, the current study confirms that the FEEL-KJ is a valuable and reliable measure of emotion regulation strategies in children and adolescents.

## Introduction

### Emotion Regulation in Children and Adolescents

Negative situations are often accompanied by feelings of anxiety, sadness, or anger. These feelings are in turn at the center of many psychiatric disorders [1]. According to functionalist perspectives on emotion, however, both positive and negative emotions are essential for human survival and should be seen as important to regulate behavior [2–5]. From this perspective, it is not the experience of negative emotions, but the inefficient regulation of these emotions that is at the basis of psychiatric symptoms [6]. Emotion regulation can be defined as *“all the extrinsic and intrinsic processes responsible for monitoring*, *evaluating and modifying emotional reactions*, *especially their intensive and temporal features*, *to accomplish one’s goal”* [7]. Theoretically, it is thus emphasized that emotion regulation strategies can focus both on maintaining or up-regulating positive emotions and on down-regulating negative emotions. In daily life, however, emotion regulation strategies generally target negative emotions [8].

Interestingly, even children at young ages are capable of regulating their emotions. A large repertoire of emotion regulation strategies can already be seen at preschool age [9] and these strategies further develop as children reach adolescence [10]. However, not all children develop the ability to effectively regulate emotions. Ineffective emotion regulation strategies were, for instance, observed in abused children [11] and in children with insecure attachment patterns [12]. The use of such maladaptive strategies has been related to emotional and behavioral maladjustment [10]. More specifically, maladaptive emotion regulation in children has been associated with depressive symptoms [13–20], anxiety symptoms [16,17,19–21], eating disorder symptoms [15], and behavioral problems [14,19,20]. Given these relations between emotion regulation and psychological health, it would be helpful to have a reliable and valid instrument to assess adaptive and maladaptive emotion regulation strategies for the age group of children and adolescents.

### Assessment of Emotion Regulation Strategies in Children and Adolescents through Self-Report

Emotion regulation strategies in children and adolescents can be measured both through other- and self-report. However, instruments that use parental or teacher reports can only assess behavior that is visible to others. Moreover, self-report measures have the advantage that they facilitate disclosing personal material. Given the sensitive and largely internal nature of emotion regulation processes, self-report therefore seems the most appropriate way to assess them [17].

Research on the assessment of emotion regulation strategies in children and adolescents through self-report was originally dominated by measures of stress coping responses. For example, based on the distinction between problem-focused and emotion-focused coping [22], and on the distinction between approach and avoidance coping [23], de Boo and Wicherts [24] developed the Coping Strategies Checklist for Children (CCSC) to measure stress coping strategies in response to ‘problems’. However, while useful, these instruments primarily assess how children and adolescents manage life problems instead of how they manage emotions. To address this, other questionnaires have been developed that focus on how children and adolescents regulate negative emotions. The Cognitive Emotion Regulation Questionnaire (CERQ), for instance, measures 5 adaptive and 4 non-adaptive cognitive emotion regulation strategies that children and adolescents use when they experience negative life events [16,25]. Similarly, the Emotion Regulation Questionnaire for Children and Adolescents (ERQ-CA) assesses the cognitive emotion regulation strategies Reappraisal and Suppression in 10- to 18-year-olds [17]. Furthermore, the Children’s Response Styles Questionnaire (CRSQ) measures how children respond to a depressed mood by tapping into the response styles Rumination, Distraction, and Problem Solving [13]. Finally, the Children’s Emotion Management Scale (CEMS) assesses the emotion regulation strategies Inhibition, Emotion Regulation Coping, and Dysregulated-Expression in response to feelings of sadness and anger [19].

However, all of the above instruments are restricted in either the number or variety of emotion regulation strategies. There is, in other words, need for an instrument that assesses a broad range of behavioral and cognitive emotion regulation strategies for several emotions in children and adolescents. The FEEL-KJ [26] is interesting in this respect because it measures 15 emotion regulation strategies for three different emotions (i.e., anxiety, sadness, and anger) and is adapted for use in both childhood and adolescence. More specifically, the FEEL-KJ assesses the emotion regulation strategies Problem Solving (e.g., “I try to change what makes me angry”), Distraction (e.g., “I do something fun”), Forgetting (e.g., “I think it will pass”), Acceptance (e.g., “I accept what makes me angry”), Humor Enhancement (e.g., “I think about things that make me happy”), Cognitive Problem Solving (e.g., “I think about what I can do”), Revaluation (e.g., “I tell myself it is nothing important”), Giving Up (e.g., “I don’t want to do anything”), Withdrawal (e.g., “I don’t want to see anyone”), Rumination (e.g., “I cannot get it out of my head”), Self-Devaluation (e.g., “I blame myself”), Aggressive Actions (e.g., “I get into a quarrel with others”), Social Support (e.g., “I tell someone how I am doing”), Expression (e.g., “I express my anger”), and Emotional Control (e.g., “I keep my feelings for myself”). Based on an exploratory factor analysis (EFA), Grob and Smolenski [26] concluded in the test manual that the first seven strategies can be classified as Adaptive Emotion Regulation and the next six strategies as Maladaptive Emotion Regulation. The remaining three strategies Social Support, Expression, and Emotional Control could not be classified as either adaptive or maladaptive. Psychometric evaluation of the FEEL-KJ in the test manual further revealed good internal consistency, good test-retest reliability, and consistent associations with external criteria such as anxiety and depression measures [26]. Interestingly, the promising external validity of the FEEL-KJ has now also received support from two other studies in which relations were shown with binge eating [15], depressive symptoms [14,15], and with the DSM symptom clusters affective problems, somatic problems, conduct problems, and ADHD problems [14].

To summarize, while the number emotion regulation instruments for children and adolescents is growing, most instruments are restricted with respect to the number or the variety of strategies they assess. The FEEL-KJ may prove to be a valuable addition to these instruments because it measures a comprehensive range of emotion regulation strategies assumed to be used by children and adolescents in response to three different emotions. Twelve of these strategies can be classified under the widely used higher order categories adaptive and maladaptive emotion regulation [6,27], making the FEEL-KJ useful for developmental psychopathology research. However, despite its promising features, the FEEL-KJ has not yet been extensively validated. For example, the internal structure of the FEEL-KJ has so far only been investigated by means of EFA [26]. It therefore remains to be tested directly to what extent the proposed two-factor model (i.e., Adaptive and Maladaptive Emotion Regulation) provides a good description of the FEEL-KJ internal structure. In addition, information on the construct validity of the FEEL-KJ is missing as it has yet to be compared to other instruments of emotion regulation. Finally, the relation of the FEEL-KJ with measures of psychopathological symptoms still has to be explored more extensively to confirm its status as a useful tool for clinical practice.

## Current Investigation

The current study attempts to validate the FEEL-KJ in a large sample of Dutch-speaking children and adolescents (N = 1102; age range = 8–18). First, the internal structure of the FEEL-KJ will be examined. To this end, it will be attempted to replicate the specified two-factor structure (Adaptive and Maladaptive Emotion Regulation) with both exploratory (EFA) and confirmatory (CFA) factor analysis.

Second, the construct and external validity of the FEEL-KJ will be examined by means of a large number of measures that were filled out by subsamples of the main sample of 1102 children and adolescents. To evaluate the construct validity of the FEEL-KJ, we will investigate its relation with the CERQ. Overall, we expect positive relations between the adaptive strategies of both instruments and between the maladaptive strategies of both instruments. In addition, we expect specific associations between six subscales of the CERQ and six subscales of the FEEL-KJ that refer to the same content, namely FEEL-KJ Self-Devaluation (e.g., “I blame myself”) and CERQ Self-Blame (e.g., “I feel that I am the one to blame for it”), FEEL-KJ Acceptance (e.g., “I accept what makes me angry”) and CERQ Acceptance (e.g., “I think that I have to accept that this has happened”), FEEL-KJ Rumination (e.g., “I cannot get it out of my head”) and CERQ Rumination (e.g., “I am preoccupied with what I think and feel about what I have experienced”), FEEL-KJ Humor Enhancement (e.g., “I think about things that make me happy”) and CERQ Positive Refocusing (e.g., “I think about pleasant experiences”), FEEL-KJ Cognitive Problem Solving (e.g., “I think about what I can do”) and CERQ Refocus on Planning (e.g., “I think of what I can do best”), FEEL-KJ Revaluation (e.g., “I tell myself it is nothing important”) and CERQ Putting into Perspective (e.g., “I tell myself there are worse things in life”).

To evaluate the external validity of the FEEL-KJ, we will calculate correlations between the FEEL-KJ and several measures of psychological well-being and distress. Based on theoretical assumptions, we expected Maladaptive Emotion Regulation to be related positively and Adaptive Emotion Regulation to be related negatively to (a) parental reports of internalizing and externalizing problems [14,19], (b) depressive symptoms [14,16–18], (c) low self-worth [13], and (d) restrictive eating disorder symptoms [15]. These measures of external validity were chosen to both replicate (depressive symptoms, internalizing problems, and externalizing problems) and extend (self-worth and restrictive eating) previous research with the FEEL-KJ.

## Method

### Participants

Data from 1118 Dutch-speaking Belgian children and adolescents was obtained for this study. Sixteen participants were excluded from the dataset. Ten participants were excluded because at least 10% of the FEEL-KJ data was missing. Four other participants were excluded because they were following special education. Finally, two participants were excluded for being older than 18. This resulted in a dataset of 1102 (443 boys, 659 girls) children and adolescents (*M*_age_ = 13.34, *SD*_age_ = 2.54, range_age_ = 8–18). Of this group, 21.9% was in elementary school, 76.3% was in secondary school, 1.5% was following higher education, and 0.4% did not indicate their level of education. Of the children in secondary school, 72.9% was following an academic track and 27.1% was learning a technical proficiency. Socioeconomic status (SES) was calculated using the Hollingshead Index based on the education and the occupation of the parents [28]. This indicated that 2.5% of the children and adolescents could be classified as ‘higher’ SES, 29.5% as ‘higher-middle’ SES, 51.5% as ‘middle SES’, 9.3% as ‘lower-middle SES’, and 2.6% as ‘lower SES’. The remaining 4.6% did not provide sufficient information to calculate SES. Please note that a subset of the present data was also recently used for different purposes [14].

### Measures

#### Child Behavior Checklist (CBCL)

The CBCL [29] is a valid and reliable questionnaire for assessing emotional and behavioral problems in children and adolescents (6–18 years old). Using parental reports on the child’s behavior in the preceding six months, it assesses symptoms of 118 different emotional and behavioral problems. Items are scored on a three-point scale ranging from 0 to 2 according to the frequency of the symptoms. This allows for the calculation of scale scores that can in turn be combined into a score on two overarching scales, namely Internalizing and Externalizing Problems. These two overarching scales represent respectively emotional and behavioral problems and will be the focus of the current study. The validity and reliability of the CBCL (*α* = 0.88 for both Internalizing and Externalizing Problems) is good and has been demonstrated across different cultures [30]. The internal consistency of the CBCL in the current study was *α* = 0.93 for Internalizing Problems and *α* = 0.86 for Externalizing Problems. No missing values were observed for the CBCL.

#### Children’s Depression Inventory (CDI)

The CDI [31] is a self-report instrument that assesses symptoms of depression in children and adolescents from 7 to 17 years old. The CDI comprises 27 items and respondents are asked to choose one out of three descriptions that fits best with how they have been feeling over the past two weeks. Responses are scored on a scale from 0 to 2. According to Kovacs [31], the reliability of the CDI in terms of internal consistency is good (α = 0.85). Cronbach’s alpha of the CDI in the current study was 0.77. Missing values were present in 0.27% of the CDI data.

#### Cognitive Emotion Regulation Questionnaire for Children (CERQ)

The CERQ [16,25] is a 36-item questionnaire that measures cognitive emotion regulation in children and adolescents. Item scores range from 1 (almost never) to 5 (almost always) and can be summed up to obtain strategy scores. The CERQ allows the calculation of nine emotion regulation strategies that can be classified as either adaptive (Acceptance, Putting into Perspective, Positive Reappraisal, Refocus on Planning, and Positive Refocusing) or non-adaptive (Catastrophizing, Rumination, Self-Blame, and Blaming Others) strategies. While the questionnaire was originally developed for individuals aged 12 and older [25], a slightly adapted version has also been validated in children under 12 [16]. The current study administered the original version of the CERQ on children aged 9 to 14 because–at the time of data collection–it was the only commercially available emotion regulation instrument in Dutch. Participants completed the CERQ in the presence of a third-year clinical psychology student and items were clarified if necessary. No problems were reported with filling out the questionnaire. According to Garnefski and colleagues [16,25], Cronbach’s alpha varies between 0.62 and 0.81 for the different subscales. Cronbach’s alpha in the current study was 0.61 for Acceptance, 0.70 for Putting into Perspective, 0.65 for Positive Reappraisal, 0.74 for Refocus on Planning, 0.85 for Positive Refocusing, 0.68 for Catastrophizing, 0.69 for Rumination, 0.62 for Self-Blame, and 0.74 for Blaming Others. Cronbach’s alpha for the higher-order scales was 0.87 for Adaptive Emotion Regulation and 0.76 for Non-Adaptive Emotion Regulation. Missing values were present in 0.31% of the CERQ data.

#### The Eating Disorder Inventory II (EDI-II)

The EDI-II [32] is a self-report questionnaire that can be administered from the age of eleven. The EDI-II was not developed to diagnose eating disorders, but rather to assess psychological characteristics common in eating disorders. The EDI-II contains 64 items that form eight subscales. Five of these subscales tap into general, personality-related characteristics of individuals with an eating disorder, but will not be used here. The remaining three subscales measure symptoms of eating disorders: Drive for Thinness, Bulimia, and Body Dissatisfaction. Subjects are asked to rate the items on a 6-point rating scale, ranging from ‘never’ to ‘always’. Total scores per subscale are obtained by summing up all item scores for the scale in question. The internal consistency of the subscales is moderate to high with Cronbach’s alpha ranging from 0.65 to 0.93 [33]. In the current study, Cronbach’s alpha was 0.91 for Drive for Thinness, 0.75 for Bulimia, and 0.92 for Body Dissatisfaction. Missing values were present in 0.34% of the EDI-II data.

#### FEEL-KJ

The FEEL-KJ [26] is a 90-item self-report measure used to assess emotion regulation strategies in response to feelings of anxiety, sadness, and anger. The original German version was developed for children and adolescents of 10 to 19 years old, but the FEEL-KJ has already been used successfully in children of 8 to 13 years old as well [15]. The questionnaire items were constructed bottom-up, based on qualitative interviews with children of 4 to 16 years old. In the final version, the authors selected 15 strategies and measured every strategy with 6 items (2 items per strategy x 3 emotions). The items are rated on a five-point scale, ranging from never to almost always. On the basis of an EFA, two orthogonal factors were distinguished: Adaptive and Maladaptive Emotion Regulation. The adaptive strategies consist of Problem Solving, Distraction, Forgetting, Acceptance, Humor Enhancement, Cognitive Problem Solving, and Revaluation. The maladaptive strategies consist of Giving Up, Withdrawal, Rumination, Self-Devaluation, and Aggressive Actions. The three remaining strategies (Expression, Social Support, and Emotional Control) could not be classified as adaptive or maladaptive. Because it is not yet clear how these remaining three strategies fit within the FEEL-KJ, they will not be used in the current study. In the test manual [26], internal consistency for the secondary scales was *α* = 0.82 for Maladaptive Emotion Regulation and *α* = 0.93 for Adaptive Emotion Regulation. Furthermore, good internal consistency was also reported for the primary scales with *α* ranging from 0.69 to 0.91. Finally, the test manual showed satisfying test-retest reliability over a six-week period and adequate correlations with symptoms of depression and anxiety. For the present study, the original German version was translated into Dutch and subsequently back-translated by a bilingual German-Dutch speaker who had experience with psychological tests. The back-translated version was consistent with the original German version of the FEEL-KJ. Examples of the Dutch FEEL-KJ items are available as Supporting Information (see S1 Appendix). Missing values were present in 0.36% of the FEEL-KJ data.

#### Self-Perception Profile for Adolescents (SPPA)

The SPPA [34] assesses the self-perception of adolescents in different areas with 5 items per scale. In the current study, we selected the scales Global Self-Worth and Physical Self-Worth. The latter was chosen because it has been evaluated as important in the studied age group [35]. To fill out the SPPA, subjects have to rate the items on a 4-point scale with higher scores reflecting greater competence. According to the test manual, the subscales have good internal consistency with Cronbach’s alpha ranging from 0.74 to 0.92 [34]. In the current study, Cronbach’s alpha was 0.85 for Physical Self-Worth and 0.77 for Global Self-Worth. Missing values were present in 0.19% of the SPPA data.

### Procedure

All participants completed the FEEL-KJ as part of two larger research projects on emotion regulation. Children and adolescents between 8 and 18 years old that were attending a regular school were eligible. To avoid overloading the children with a large battery of tests, only the FEEL-KJ was completed by all children. The remaining tests were filled out by subsets of the total sample. The institutional review board of Ghent University approved the protocols of both projects. Written informed consent was acquired from all participating children and from their parents. No reward was given for participation.

First, children from different classes of multiple secondary schools in a large Belgian city (i.e., Antwerp) were contacted, resulting in 424 participants (269 girls and 155 boys, *M*_age_ = 14.91, *SD*_age_ = 1.63, range_age_ = 11–18) that filled out the FEEL-KJ during one class hour supervised by a psychologist. In addition to the FEEL-KJ, all children of the first recruitment wave filled out the EDI-II and the SPPA in a random order. One child did not fill out the EDI-II, resulting in a sample of 423 children and adolescents for the EDI-II.

However, because the participating schools were mainly situated in urban areas, we were concerned with an overrepresentation of children living in the city at the expense of youth from more rural areas. To address this issue, we instructed third-year clinical psychology students living all over Flanders to recruit participants in their near home environment. In contrast to the first recruitment wave, children in the second recruitment wave completed the FEEL-KJ at home under the supervision of the psychology student. This recruitment method resulted in an additional 678 children (390 girls and 288 boys, *M*_age_ = 12.37, *SD*_age_ = 2.52, range_age_ = 8–18). In addition to the FEEL-KJ, 191 children (104 girls and 87 boys, *M*_age_ = 11.75, *SD*_age_ = 1.30, range_age_ = 9–14) of the second recruitment wave filled out the CERQ and the CDI in a random order. Furthermore, 99 children (61 girls, 38 boys, *M*_age_ = 12.14, *SD*_age_ = 1.39, range_age_ = 10–15) completed the FEEL-KJ a second time exactly two weeks after the first test moment. Finally, the parents of 428 children (255 girls and 173 boys, *M*_age_ = 12.06, *SD*_age_ = 1.83, range_age_ = 8–18) recruited in the second wave filled out the CBCL.

### Data Analysis

All analyses were performed with SPSS 22 for Windows, except for the CFA which was performed in R [36] with the lavaan package [37]. Expectation-maximization was used to provide estimates of the missing values. Unless stated otherwise, correlation coefficients represent Pearson product-moment correlation coefficients.

A significance level of α = 0.05 will be applied throughout the manuscript. However, because meaningless correlations can sometimes become significant in large samples, only significant correlations with a correlation coefficient ≥ 0.10 will be interpreted. According to Cohen’s rules of thumb [38], a correlation coefficient of 0.10 corresponds to a small effect size. The complete dataset is available as Supporting Information (see S1 Dataset).

## Results

### Descriptive Statistics

The FEEL-KJ data (Table 1) covered the entire range of scores (i.e., 6–30) for all primary scales. Mean scores ranged from 16.73 (Revaluation) to 21.11 (Humor Enhancement) for the adaptive strategies and from 12.83 (Aggressive Actions) to 17.45 (Rumination) for the maladaptive strategies. In line with this pattern, the score distribution of the adaptive strategies displayed a mild negative skewness with skewness scores ranging from -0.45 (Humor Enhancement) to 0.09 (Revaluation), whereas the score distribution of the maladaptive strategies displayed a mild positive skewness with skewness scores ranging from 0.03 (Rumination) to 0.69 (Aggressive Actions). Kurtosis scores ranged from -0.33 (Distraction) to 0.28 (Forgetting) for the adaptive strategies and from -0.06 (Rumination) to 0.12 (Aggressive Actions) for the maladaptive strategies. QQ-plots indicated that the score distribution of the different strategies did not deviate substantially from normality.

**Table 1:** FEEL-KJ properties.

	Mean (SD)	Skewness	Kurtosis	α	r_t1-t2_
**Problem Solving**	20.19 (4.48)	-0.33	0.25	0.75	0.85
**Distraction**	21.01 (5.48)	-0.44	-0.34	0.87	0.88
**Forgetting**	19.68 (4.29)	-0.29	0.28	0.71	0.76
**Acceptance**	19.13 (4.30)	-0.10	0.03	0.72	0.81
**Humor Enhancement**	21.11 (5.39)	-0.45	-0.28	0.86	0.88
**Cognitive Problem Solving**	19.36 (4.83)	-0.34	0.14	0.81	0.86
**Revaluation**	16.73 (4.82)	0.09	0.01	0.79	0.87
**Giving Up**	14.31 (4.50)	0.38	-0.03	0.72	0.82
**Withdrawal**	13.94 (4.80)	0.46	0.10	0.76	0.79
**Rumination**	17.45 (4.37)	0.03	-0.06	0.64	0.84
**Self-Devaluation**	15.95 (4.58)	0.27	0.02	0.75	0.83
**Aggressive Actions**	12.83 (5.12)	0.69	0.12	0.83	0.78

**Note.** N = 1102, except for test-retest reliability (N = 99).

### Internal Structure

To investigate the internal structure of the FEEL-KJ, we attempted to replicate previous reports of an orthogonal two-factor structure (Adaptive and Maladaptive Emotion Regulation) with both EFA and CFA [26]. A principal component analysis (PCA) revealed two components with an eigenvalue above the cut-off value of one (4.47 and 2.65), suggesting a two-factor structure for the EFA. This was supported by the scree plot, which showed a clear elbow after the first two components. Together, these two components explained 59.29% of the variance. Based on the above, a principal axis EFA with two factors was computed and subjected to an oblimin rotation. The rotated sums of the squared loadings for the two factors were 4.46 and 2.65 respectively. In line with previous research [26], no substantial correlation was observed between the two factors (r = -0.001). As can be seen in Table 2, the first factor represents the adaptive strategies, whereas the second factor represents the maladaptive strategies.

**Table 2:** Factor loadings of the FEEL-KJ exploratory factor analysis.

	Adaptive Emotion Regulation	Maladaptive Emotion Regulation
**Problem Solving**	0.86	-0.05
**Distraction**	0.83	-0.21
**Forgetting**	0.74	0.10
**Acceptance**	0.76	-0.14
**Humor Enhancement**	0.84	-0.14
**Cognitive Problem Solving**	0.71	0.26
**Revaluation**	0.72	0.15
**Giving Up**	-0.26	0.76
**Withdrawal**	-0.18	0.74
**Rumination**	0.20	0.74
**Self-Devaluation**	0.24	0.69
**Aggressive Actions**	-0.02	0.56

**Note.** N = 1102.

To test how well the orthogonal two-factor model (model 1) described the internal structure of the FEEL-KJ, we performed a CFA with robust maximum likelihood estimation on the variance-covariance matrix. Goodness-of-fit was assessed by evaluating the Satorra-Bentler scaled chi-square, the Comparative Fit Index (CFI), the Root Mean Square Error of Approximation (RMSEA), and the Standardized Root Mean Square Residual (SRMR). According to Schermelleh-Engel, Moosbrugger, and Müller [39], a CFI of 0.97 ≤ CFI ≤ 1.00 indicates a good fit and a CFI of 0.95 ≤ CFI < 0.97 indicates an acceptable fit. Furthermore, a RMSEA of 0 ≤ RMSEA ≤ 0.05 indicates a good fit and a RMSEA of 0.05 < RMSEA ≤ 0.08 indicates an acceptable fit. Finally, a SRMR of 0 ≤ SRMR ≤ 0.05 indicates a good fit and a SRMR of 0.05 < SRMR ≤ 0.10 indicates an acceptable fit. The CFA of model 1 revealed that the model provided a poor fit of the data, χ^2^(54) = 948.19, *p* < 0.001, CFI = 0.80, RMSEA = 0.12, SRMR = 0.11. To investigate the cause of the misfit, the dataset was first split into two groups. This allowed us to improve the model on the basis of the data from one group (group A) and to validate the improved model on the data from the other group (group B). Note that the dataset was split into two groups separately for each combination of gender and age to ensure that the two groups were equal with respect to age and gender. When a certain age-gender combination contained an odd number of members, the remaining member was randomly assigned to one of the two groups. This procedure resulted in 551 members in both group A and group B.

In line with the results obtained for the entire dataset, model 1 did not provide a good fit for group A, χ^2^(54) = 493.02, *p* < 0.001, CFI = 0.80, RMSEA = 0.12, SRMR = 0.11. A look at the correlation matrix of group A (Table 3) revealed that the correlations between the different adaptive strategies and the correlations between the different maladaptive strategies were consistent. It also revealed, however, that three separate groups of maladaptive strategies could be distinguished on the basis of their relation with the adaptive strategies. More specifically, it was found that Withdrawal and Giving up displayed a negative correlation with the adaptive strategies, Rumination and Self-Devaluation displayed a positive correlation with the adaptive strategies, and Aggressive Actions displayed no correlation with the adaptive strategies. The correlation matrix suggested, in other words, that the Maladaptive Emotion Regulation factor should be subdivided into three lower-level factors based on their correlation with the adaptive strategies. In line with their content, these factors were named Avoidance (Withdrawal and Giving Up), Dysfunctional Thoughts (Rumination and Self-Devaluation), and Aggression (Aggressive Actions).

**Table 3:** FEEL-KJ correlation matrix.

	1	2	3	4	5	6	7	8	9	10	11	12
**1. Problem Solving**	–	0.70***	0.58***	0.65***	0.71***	0.57***	0.53***	-0.24***	-0.19***	0.10*	0.10*	-0.03
**2. Distraction**	0.63***	–	0.50***	0.65***	0.83***	0.43***	0.49***	-0.33***	-0.29***	-0.04	0.00	-0.05
**3. Forgetting**	0.59***	0.50***	–	0.53***	0.54***	0.42***	0.62***	-0.02	0.00	0.18***	0.18***	0.00
**4. Acceptance**	0.62***	0.56***	0.50***	–	0.61***	0.40***	0.53***	-0.26***	-0.19***	-0.02	0.11**	-0.01
**5. Humor Enhancement**	0.66***	0.80***	0.50***	0.49***	–	0.46***	0.52***	-0.24***	-0.22***	0.05	0.05	-0.08
**6. Cognitive Problem Solving**	0.60***	0.47***	0.40***	0.43***	0.53***	–	0.48***	-0.06	0.01	0.42***	0.29***	0.04
**7. Revaluation**	0.48***	0.43***	0.59***	0.39***	0.46***	0.48***	–	-0.07	-0.06	0.17***	0.23***	0.06
**8. Giving Up**	-0.22***	-0.32***	-0.13**	-0.29***	-0.26***	-0.07	-0.07	–	0.60***	0.44***	0.35***	0.34***
**9. Withdrawal**	-0.13**	-0.26***	0.00	-0.21***	-0.20***	-0.02	-0.02	0.57***	–	0.37***	0.38***	0.33***
**10. Rumination**	0.13**	0.05	0.10*	0.03	0.09*	0.40***	0.17***	0.36***	0.32***	–	0.48***	0.32***
**11. Self-Devaluation**	0.17***	0.08	0.19***	0.10*	0.08	0.29***	0.26***	0.35***	0.40***	0.47***	–	0.23***
**12. Aggressive Actions**	0.00	-0.05	-0.07	-0.08*	-0.08	0.08	0.02	0.35***	0.26***	0.35***	0.22***	–

Note.

*p < 0.05

**p < 0.01

***p < 0.001.

Values below the diagonal represent correlation coefficients in group A (N = 551), whereas values above the diagonal represent correlation coefficients in group B (N = 551).

**Fig 1:**
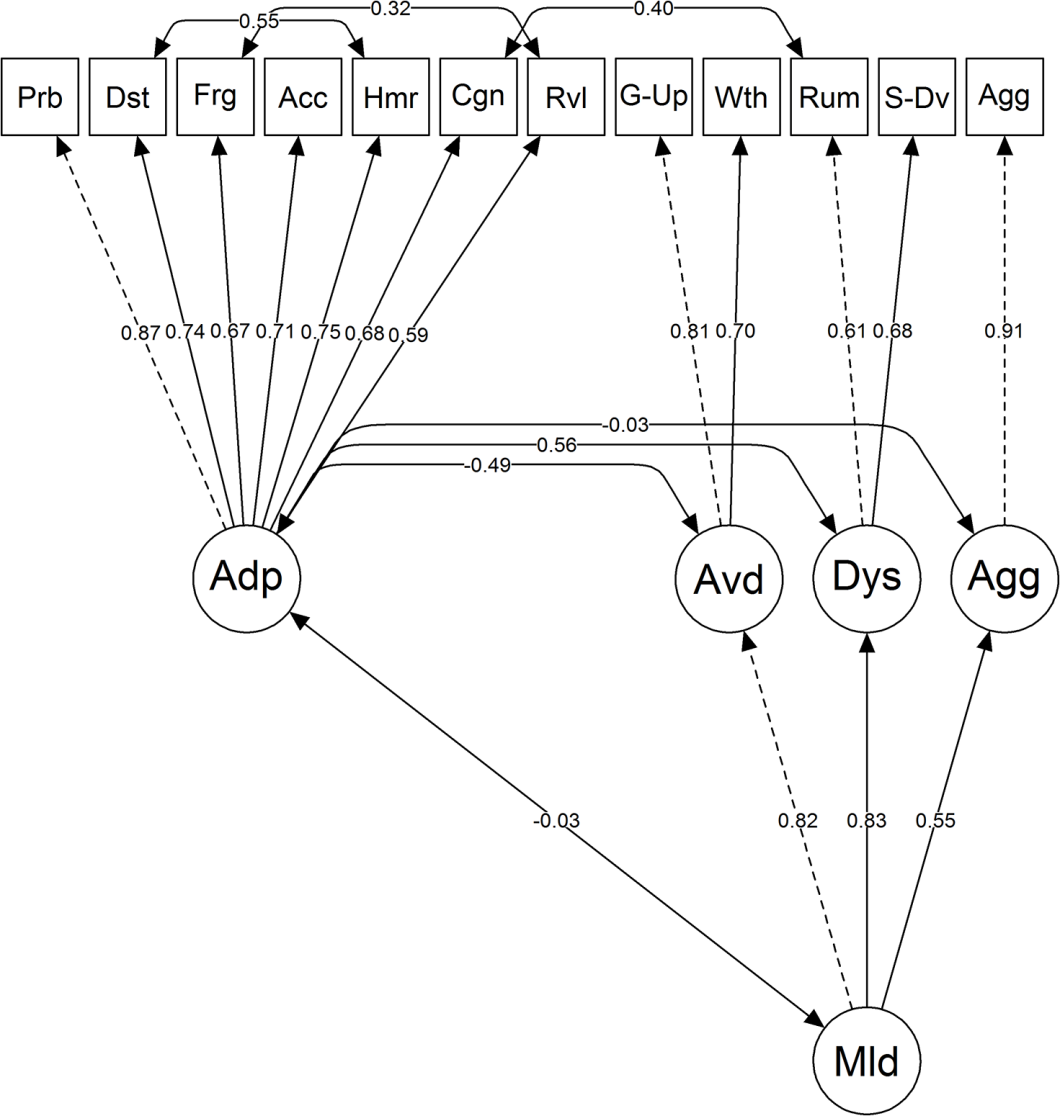
A visual representation of model 3. One-sided arrows reflect standardized factor loadings for group A (N = 551). Double-sided arrows reflect modeled correlation coefficients for group A. Dotted lines reflect fixed values. Squares represent manifest variables and circles represent latent variables. Legend: Prb = Problem Solving, Dst = Distraction, Frg = Forgetting, Acc = Acceptance, Hmr = Humor Enhancement, Cgn = Cognitive Problem Solving, Rvl = Revaluation, G-Up = Giving Up, Wth = Withdrawal, Rum = Rumination, S-Dv = Self-Devaluation, Agg (manifest) = Aggressive Actions, Adp = Adaptive Emotion Regulation, Avd = Avoidance, Dys = Dysfunctional Thoughts, Agg (latent) = Aggression, Mld = Maladaptive Emotion Regulation.

To test the revised two-factor model with three lower-level maladaptive factors (model 2), we subjected it to a CFA. Note that the variance of the Aggressive Actions scale was fixed to (1 – α)*variance to account for the imperfect reliability of the scale scores [40]. In addition, note that a restriction was imposed on the relation between the higher-order factors Adaptive Emotion Regulation and Maladaptive Emotion Regulation in order to make the model identifiable. More specifically, the model was estimated under the restriction that the covariance between the higher-order factors Adaptive Emotion Regulation and Maladaptive Emotion Regulation should be equal to the mean of the covariances between the higher-order factor Adaptive Emotion Regulation and the lower-order factors Avoidance, Dysfunctional Thoughts, and Aggression. Model 2 proved to be a significant improvement over model 1, χ^2^(5) = 129.56, *p* < 0.001, but still did not provide an acceptable fit for the data, χ^2^(49) = 359.82, *p* < 0.001, CFI = 0.86, RMSEA = 0.11, SRMR = 0.07. A subsequent inspection of the modification indices revealed that this was in large part due to residual covariances between a number of strategies with similar content, namely Distraction and Humor Enhancement, Forgetting and Revaluation, and Cognitive Problem Solving and Rumination. Adding these relations to the model (model 3; Fig 1) significantly improved its fit, χ^2^(3) = 189.96, *p* < 0.001, to an acceptable level, χ^2^(46) = 158.20, *p* < 0.001, CFI = 0.95, RMSEA = 0.07, SRMR = 0.06.

Finally, model 3 was tested on group B as a means of cross-validation. The results of the CFA, χ^2^(46) = 186.61, *p* < 0.001, indicated that the RMSEA (0.07) and SRMR (0.06) were acceptable, whereas the CFI (0.94) was just below the cut-off value for acceptability. Given the fact that the fit indices were acceptable or near-acceptable and given the fact that they were very similar to the fit indices obtained for group A, it can be concluded that model 3 also provided an adequate fit for group B.

### Reliability

Reliability of the FEEL-KJ was assessed by calculating Cronbach’s α-coefficient for each scale. Alpha coefficients ranged from 0.64 (Rumination) to 0.87 (Distraction) for the primary scales (Table 1). For the higher-order scales, internal consistency was 0.94 for Adaptive Emotion Regulation and 0.86 for Maladaptive Emotion Regulation.

Additionally, test-retest reliability (Table 1) was tested within a time interval of two weeks. For the primary scales, test-retest correlation coefficients ranged from 0.76 (Forgetting) to 0.88 (Humor Enhancement). Test-retest correlation coefficients for the higher-order scales were 0.90 for Adaptive Emotion Regulation and 0.88 for Maladaptive Emotion Regulation.

### Construct Validity

To evaluate the construct validity of the FEEL-KJ, we first calculated correlations between the FEEL-KJ higher-order scales (Adaptive and Maladaptive Emotion Regulation) and the CERQ higher-order scales (Adaptive and Non-Adaptive Emotion Regulation). For FEEL-KJ Adaptive Emotion Regulation, a strong positive relation was found with CERQ Adaptive Emotion Regulation, *r* = 0.67, *p* < 0.001, and a weak positive relation was found with CERQ Non-Adaptive Emotion Regulation, *r* = 0.16, *p* = 0.032. For FEEL-KJ Maladaptive Emotion Regulation, on the other hand, a positive relation was found with CERQ Non-Adaptive Emotion Regulation, *r* = 0.36, *p* < 0.001, and no relation was found with CERQ Adaptive Emotion Regulation, *r* = -0.11, *p* = 0.123.

Next, to investigate the predicted relations between the FEEL-KJ and CERQ primary scales, we calculated the partial correlation between each FEEL-KJ subscale and the relevant CERQ subscales while controlling for the effect of the remaining FEEL-KJ subscales (Table 4). This analysis confirmed five of the six expected relations: (1) CERQ Self-Blame was primarily related to FEEL-KJ Self-Devaluation, *r* = 0.35, *p* < 0.001, (2) CERQ Acceptance was only related to FEEL-KJ Acceptance, *r* = 0.19, *p* = 0.009, (3) CERQ Rumination was associated primarily with FEEL-KJ Rumination, *r* = 0.21, *p* = 0.005, (4) CERQ Positive Refocusing was related mainly to FEEL-KJ Humor Enhancement, *r* = 0.31, *p* < 0.001, and (5) CERQ Refocus on Planning was mainly associated with FEEL-KJ Cognitive Problem Solving, *r* = 0.37, *p* < 0.001. In contrast to our expectations, CERQ Putting into Perspective was not associated with FEEL-KJ Revaluation, *r* = 0.03, *p* = 0.735, but with FEEL-KJ Humor Enhancement, *r* = 0.15, *p* = 0.045, and FEEL-KJ Giving Up, *r* = -0.16, *p* = 0.031.

**Table 4:** Partial correlations between FEEL-KJ (rows) and CERQ (columns) primary scales.

	Self-Blame	Acceptance	Rumination	Positive Refocusing	Refocus on Planning	Putting into Perspective
**Problem Solving**	-0.13	0.11	0.07	0.10	0.08	0.03
**Distraction**	-0.03	-0.06	-0.04	0.12	0.01	0.10
**Forgetting**	-0.01	-0.03	-0.11	0.10	-0.08	0.11
**Acceptance**	0.03	0.19**	0.08	-0.08	-0.03	0.00
**Humor Enhancement**	0.17*	0.07	0.13	0.31***	0.18*	0.15*
**Cognitive Problem Solving**	0.11	-0.06	0.14	0.00	0.37***	-0.13
**Revaluation**	0.02	0.10	0.06	0.06	0.12	0.03
**Giving Up**	-0.11	0.12	0.12	-0.14	-0.09	-0.16*
**Withdrawal**	0.04	0.03	0.03	0.09	0.08	0.02
**Rumination**	0.04	0.09	0.21**	-0.04	-0.02	0.12
**Self-Devaluation**	0.35***	0.00	0.17*	-0.15*	-0.08	0.06
**Aggressive Actions**	0.10	-0.08	-0.02	0.05	0.09	0.07

**Note.** N = 191

*p < 0.05

**p < 0.01

***p < 0.001.

### External Validity

#### General Psychopathology

FEEL-KJ Adaptive Emotion Regulation correlated negatively with both CBCL Internalizing Problems, *r* = -0.14, *p* = 0.004, and CBCL Externalizing Problems, *r* = -0.11, *p* = 0.029. FEEL-KJ Maladaptive Emotion Regulation, on the other hand, did not correlate significantly with either CBCL Internalizing Problems, *r* = 0.02, *p* = 0.643, or CBCL Externalizing Problems, *r* = 0.05, *p* = 0.293.

#### Depressive Symptoms

CDI scores were found to correlate negatively with FEEL-KJ Adaptive Emotion Regulation, *r* = -0.40, *p* < 0.001, but positively with FEEL-KJ Maladaptive Emotion Regulation, *r* = 0.35, *p* < 0.001.

#### Self-perception

FEEL-KJ Adaptive Emotion Regulation was related positively to SPPA Global Self-Worth, *r* = 0.27, *p* < 0.001, and SPPA Physical Self-Worth, *r* = 0.20, *p* < 0.001, whereas FEEL-KJ Maladaptive Emotion Regulation was related negatively to SPPA Global Self-Worth, *r* = -0.50, *p* < 0.001, and SPPA Physical Self-Worth, *r* = -0.44, *p* < 0.001.

#### Eating Disorder Symptoms

FEEL-KJ Adaptive Emotion Regulation correlated negatively with the three included EDI-II subscales Bulimia, *r* = -0.16, *p* = 0.001, Body Dissatisfaction, *r* = -0.17, *p* = 0.001, and Drive for Thinness, *r* = -0.13, *p* = 0.006. FEEL-KJ Maladaptive Emotion Regulation, on the other hand, correlated positively with Bulimia, *r* = 0.49, *p* < 0.001, Body Dissatisfaction, *r* = 0.45, *p* < 0.001, and Drive for Thinness, *r* = 0.39, *p* < 0.001.

## Discussion

Although the field of emotion regulation research in children and adolescents is growing, there is need for adequate instruments that cover a wide range of emotion regulation strategies. A promising instrument in this respect is the FEEL-KJ [26] because it measures 15 emotion regulation strategies in response to different emotions. In the present study, we evaluated the internal structure, reliability, and validity of the FEEL-KJ as an instrument to measure emotion regulation strategies in children and adolescents.

### Internal Structure of the FEEL-KJ

The investigation of the internal structure confirmed earlier reports of a two-factor structure with Adaptive Emotion Regulation and Maladaptive Emotion Regulation as overarching categories [26]. This finding is in line with the notion that emotion regulation strategies in both adults [6,25,27] and children [10,14,16–18,26] can be classified in terms of their influence on psychological health. However, the structure of the two-factor model appeared to be more complex than what was previously assumed. That is, the results indicated that the Maladaptive Emotion Regulation factor should be subdivided into three lower-level factors (i.e., Avoidance, Dysfunctional Thoughts, and Aggression) on the basis of their relation with the Adaptive Emotion Regulation factor. These lower-level factors correspond to previous distinctions that have been made in the literature. More precisely, two of them reflect approach behavior (i.e., Dysfunctional Thoughts and Aggression) and one reflects avoidance behavior (i.e., Avoidance) [23]. In addition, the approach factors can be further categorized as reflecting cognitive (i.e., Dysfunctional Thoughts) and behavioral (i.e., Aggression) emotion regulation [25]. As can be expected from relations between maladaptive and adaptive strategies, Avoidance correlated negatively and Aggression did not correlate with Adaptive Emotion Regulation. Dysfunctional Thoughts, on the other hand, correlated positively with Adaptive Emotion Regulation. A possible explanation for this positive relation is that Rumination and Self-Devaluation are often a reaction to the failure of adaptive emotion regulation strategies. From a process perspective on emotion regulation [41], it can be argued that the first response to negative emotions is to try and solve the problem that caused the emotions (i.e., Problem Solving and Cognitive Problem Solving). Alternatively, if the child is not skilled at problem solving or if the negative emotions are overwhelming, the first response can be to close down (i.e., Giving Up and Withdrawal) or to react aggressively (i.e., Aggressive Actions). If the problem proves to be persistent, an adaptive emotion regulation response may then be to focus on strategies whose aim is to attenuate the intensity of the negative emotions (i.e., Distraction, Forgetting, Humor Enhancement, and Revaluation). However, if these strategies fail to attenuate these emotions, it is reasonable to assume that the negative emotions start to control the mind of the child (i.e., Rumination) and that the child thinks it is his/her own fault that these emotions persist (i.e., Self-Devaluation). In this line of reasoning, Dysfunctional Thoughts and Adaptive Emotion Regulation may be related positively because the former is a common response to the failure of the latter.

In addition to the increased complexity of the factor structure, residual correlations between Distraction and Humor Enhancement, Forgetting and Revaluation, and Cognitive Problem Solving and Rumination had to be included in the model. This is not surprising, given the highly similar content of these strategies. Distraction (e.g., “I do something fun”) and Humor Enhancement (e.g., “I think about things that make me happy”) both refer to emotion regulation strategies whose aim is to up-regulate positive emotions. Furthermore, Forgetting (e.g., “I think it will pass”) and Revaluation (“I tell myself it is nothing important”) both aim to attenuate the emotional impact of a negative event. Finally, Cognitive Problem Solving (e.g., “I think about what I could do”) and Rumination (e.g., “I cannot get it out of my head”) both refer to strategies that involve thinking about the event that caused the negative emotions.

### Reliability and Validity of the FEEL-KJ

Reliability of the FEEL-KJ proved to be good with internal consistency above α = 0.70 except for Rumination (α = 0.64) and test-retest reliability above *r* = 0.75. This closely replicates the original evaluation of the FEEL-KJ [26] and is in line with other coping [24,42] and emotion regulation [13,16,17,19,25] instruments.

Construct validity of the FEEL-KJ was evaluated by calculating correlations between the FEEL-KJ and the CERQ. In line with the expectations, the closest relations were found between FEEL-KJ Adaptive Emotion Regulation and CERQ Adaptive Emotion Regulation and between FEEL-KJ Maladaptive Emotion Regulation and CERQ Non-Adaptive Emotion Regulation. Interestingly, further investigation revealed that the relation between the subscales of the CERQ and the FEEL-KJ was strongest for subscales with similar content. More precisely, it was found that CERQ Self-Blame was related primarily to FEEL-KJ Self-Devaluation, CERQ Acceptance to FEEL-KJ Acceptance, CERQ Rumination to FEEL-KJ Rumination, CERQ Positive Refocusing to FEEL-KJ Humor Enhancement, and CERQ Refocus on Planning to FEEL-KJ Cognitive Problem Solving. However, despite their overlap in content, no relation was found between CERQ Putting into Perspective and FEEL-KJ Revaluation. While both scales measure the extent to which a negative situation is minimized, it is possible that the absence of a relation between the scales reflects the fact that they measure different ways of problem minimization. That is, while CERQ Putting into Perspective measures a relative approach to problem minimization (e.g., “I tell myself there are worse things in life), FEEL-KJ Revaluation measures an absolute approach (e.g., “I tell myself it is nothing important”). These two approaches may rely on different cognitive processes, explaining why no correlation was found between the two emotion regulation strategies. In support of this view, CERQ Putting into Perspective was found to be related to FEEL-KJ Humor Enhancement. Similar to CERQ Putting into Perspective, this strategy aims to reduce the impact of a negative event by thinking about another situation (e.g., “I think about things that make me happy”).

External validity of the FEEL-KJ was assessed by calculating correlations between the FEEL-KJ higher-order scales and four different indicators of psychological well-being and distress, namely general psychopathology (CBCL), depressive symptoms (CDI), eating disorder symptoms (EDI-II), and self-perception (SPPA). In general, FEEL-KJ Adaptive Emotion Regulation was found to be correlated negatively with measures of psychological distress and positively with measures of psychological well-being, whereas FEEL-KJ Maladaptive Emotion Regulation was found to be correlated positively with measures of psychological distress and negatively with measures of psychological well-being. These findings confirm previous research on the FEEL-KJ in which relations were found with depressive symptoms [14,15,26] and parental reports of externalizing and internalizing problems [14]. In addition, and in line with other research, these findings also extend the predictive value of the FEEL-KJ to self-worth [13] and restrictive eating disorder symptoms [15].

Interestingly, a distinction can be made between the relations with self-report measures (CDI, EDI-II, SPPA) and the relations with other-report measures (CBCL) of psychological well-being and distress. That is, relations with other-report measures were less consistent and substantially smaller than relations with self-report measures. This is in line with earlier research that has related both self- and other-report measures of psychopathology to emotion regulation [19]. Because the FEEL-KJ is a self-report measure, a likely reason for this pattern is that it is due to informant differences [19,43]. Because various informants may be complementary in providing crucial data concerning the child’s psychosocial functioning, the use of multiple informants in child psychosocial assessment is broadly accepted and recommended in literature [44].

### Strengths and Limitations of the FEEL-KJ

An important asset of the FEEL-KJ is that it is constructed bottom-up instead of top-down. Because of this, the FEEL-KJ is not restricted to emotion regulation strategies that are part of a specific theoretical framework but instead includes a wide range of strategies children and adolescents commonly use. This allows clinical practitioners to get a detailed overview of the emotion regulation strategies that are used by a specific child or adolescent. Consequently, the FEEL-KJ is a valuable tool to identify the strengths and weaknesses of a child as part of a larger psychological assessment process. When using the FEEL-KJ for purposes of clinical assessment, clinicians should keep in mind two important points. First, they should not only look out for the excessive use of maladaptive strategies but also for the limited use of adaptive strategies because both were found to be associated with psychological distress. Second, they should pay attention to the flexibility with which children and adolescents deploy emotion regulation strategies. To this end, it is useful to examine the range of emotion regulation strategies that are at the disposal of the child. Specifically, it can be expected that children with a limited emotion regulation repertoire are likely to experience emotional distress in situations where the available emotion regulation strategies have failed or cannot be applied.

If the FEEL-KJ reveals weaknesses in emotion regulation, it seems useful to also administer the Difficulties in Emotion Regulation Scale (DERS) [45] as this instrument was developed to measure the underlying processes that result in problems with emotion regulation (e.g., ‘Lack of Emotional Awareness, ‘Limited Access to Emotion Regulation Strategies’). The DERS thus complements the FEEL-KJ with information on possible underlying difficulties in dealing with emotions. Once these problems have been addressed, cognitive behavioral techniques can be applied to reduce the use of maladaptive strategies and increase the use of adaptive strategies based on the emotion regulation profile derived from the FEEL-KJ. The adaptive strategies Acceptance, Problem Solving and Revaluation are especially interesting in this respect, since previous research has identified them as important transdiagnostic tools in the treatment of psychopathology in both adults and children [6,46].

The properties of the FEEL-KJ also make it an interesting tool for research. Research with the FEEL-KJ can focus both on the primary scales and on the higher-order scales. When one does not have hypotheses regarding specific emotion regulation strategies, it is recommended to use the higher-order scales as these are more comprehensive and more reliable than the primary scales. Nevertheless, the wide range of strategies covered by the FEEL-KJ also allows to investigate the influence of specific emotion regulation strategies. The FEEL-KJ can, for instance, be used to test whether different emotion regulation strategies relate to different psychological problems [14]. In addition, it can also be used to compare the usefulness of different adaptive strategies as a buffer or treatment for psychological problems. Finally, the FEEL-KJ can be used to test, extend, and compare theories of psychopathology. For example, the Response Style Theory posits that the severity and intensity of depressive symptoms is determined by three important response styles, namely Rumination, Problem Solving, and Distraction [13]. The FEEL-KJ can provide a stringent test of this theory because it does not only include Rumination, Problem Solving, and Distraction as emotion regulation strategies but also 12 other strategies. The FEEL-KJ can, in other words, be used to test if the strategies Rumination, Problem Solving, and Distraction are stronger predictors of depressive symptoms than the other strategies that are included in the questionnaire.

However, despite the abovementioned strengths, the current study also reveals some limitations of the FEEL-KJ. One limitation is that it is not yet clear how the strategies Expression, Social Support, and Emotional Control fit within the FEEL-KJ structure. One possible explanation is that these strategies cannot be classified as either adaptive or maladaptive because their effectiveness depends largely on the context in which they are deployed. The outcome of seeking social support could, for example, depend on whether or not the feelings of the child are taken seriously by its surrounding. Before Expression, Social Support, and Emotional Control are incorporated in the FEEL-KJ, future research should investigate their informative value by studying how they relate to psychological health in different contexts.

Another limitation is that the FEEL-KJ has yet to be evaluated in clinical groups. That is, to further establish the validity of the FEEL-KJ, future research should investigate whether children with diagnosed psychopathology employ more maladaptive and less adaptive strategies than control children. In addition, to get a detailed overview of the relation between the FEEL-KJ and psychological distress, future research should examine whether the different FEEL-KJ emotion regulation strategies are related to specific psychopathologies or whether they are related transdiagnostically to psychopathology [6]. Finally, future studies should aim to extend the cross-sectional nature of the current study to longitudinal designs for a better understanding of the long-term relations between FEEL-KJ emotion regulation strategies and psychopathology.

To conclude, the current study confirms the reliability and validity of the FEEL-KJ in a large sample of children and adolescents. Furthermore, evidence was acquired for a two-factor structure in which 7 strategies can be classified as Adaptive Emotion Regulation and 5 strategies can be classified as Maladaptive Emotion Regulation. While further examination of the FEEL-KJ is recommended, the present investigation supports its relevance for both clinical practice and research.

## Supporting Information

S1 Appendix Dutch FEEL-KJ example items adapted from "FEEL-KJ Vragenlijst over emotieregulatie bij kinderen en jongeren" with permission from Hogrefe Uitgevers BV. (PDF)

S1 Dataset Complete dataset used for the current study. (ZIP)
