# Correction: Validation of the FEEL-KJ: An Instrument to Measure Emotion Regulation Strategies in Children and Adolescents

**DOI:** 10.1371/journal.pone.0144324

**Published:** 2015-12-02

**Authors:** The PLOS ONE Staff

## Notice of Republication

S1 Appendix was published in error. The error was corrected in the HTML version of this article on November 12, 2015, and the corrected appendix is available in the supporting information of this article.

## Supporting Information

S1 File Corrected S1 Appendix. (PDF)
