# Supplementary material for: Validation of the FEEL-KJ: An Instrument to Measure Emotion Regulation Strategies in Children and Adolescents
Source: PLoS One. 2015 Sep 2;10(9):e0137080. doi: 10.1371/journal.pone.0137080 (PMC4557990; doi:10.1371/journal.pone.0137080)
Supplement: S1 Appendix — (PDF) [file pone.0137080.s001.pdf]

S1 Appendix: Dutch FEEL-KJ example items

Below are five example items of the Dutch FEEL-KJ. For the full set of items, please contact Hogrefe Publishers at info@hogrefe.nl.

Dutch FEEL-KJ example items for anger. Items are preceded by an introduction and the following sentence: 'Wanneer ik boos ben …'

**Problem Solving**	Probeer ik dat wat me boos maakt te veranderen.
**Distraction**	Doe ik iets wat ik leuk vind.
**Humor Enhancement**	Denk ik aan dingen die me gelukkig maken.
**Withdrawal**	Trek ik me terug.
**Rumination**	Krijg ik het maar niet uit mijn hoofd.

Note. Items are similar for anxiety and sadness (total N = 90).
